# Forensic appraisal of death due to acute alcohol poisoning: three case reports and a literature review

**DOI:** 10.1080/20961790.2019.1572259

**Published:** 2019-03-18

**Authors:** Hui Wang, Hongmei Xu, Wencan Li, Beixu Li, Qun Shi, Kaijun Ma, Bi Xiao, Long Chen

**Affiliations:** a Department of Forensic Medicine, School of Basic Medical Sciences, Fudan University, Shanghai, China; b Institute of Criminal Scientific Technology, Shanghai Public Security Bureau, Pudong Branch, Shanghai, China; c Forensic Laboratory, Criminal Science and Technology Institute, Shanghai Public Security Bureau, Shanghai, China

**Keywords:** Forensic sciences, forensic pathology, acute alcohol poisoning, blood alcohol concentration, central nervous system depression

## Abstract

Death due to acute alcohol poisoning lacks specific anatomical characteristics, compared with other deaths due to drug poisoning. We report three forensic cases of death from acute alcohol poisoning due to inhibition of the respiratory centre and eventual asphyxia. Blood alcohol concentrations in the three fatalities were 5.28, 3.33 and 3.78 mg/mL, respectively. Lethal doses and blood alcohol concentrations showed differences between individuals. Detailed auxiliary tests besides autopsy were undertaken. These cases show that forensic scientists should exclude other causes of death, combine the autopsy with auxiliary tests, and then make an appraisal.

## Introduction

Alcohol is the psychoactive substance encountered most often in forensic toxicology [1]. Moderate consumption of alcohol can reduce the risk of cardiovascular diseases and type 2 diabetes mellitus [2], contribute to formation of positive and optimistic lifestyles, and improve quality of life [3]. However, alcohol abuse can trigger alcohol-related diseases, a trend of consuming alcohol at a younger age, and can lead to violent behaviour [4–7]. At high blood alcohol concentrations (BACs; >4 mg/mL) people are likely to harm themselves or suffer blunt trauma through accidental falls [8].

Alcohol abuse can also cause death. According to World Health Organization reports, ∼3.3 million people worldwide die due to alcohol consumption each year, which accounted for 5.9% of all deaths in 2012 [9]. Excessive consumption of alcohol can cause death by drowning, traffic accidents or violence. A study in Slovakia showed that death due to acute alcohol poisoning (AAP) accounts for a significant proportion of all deaths related to alcohol consumption [10]. Similar results have been found in women [11]. Death due to AAP is a serious consequence of heavy drinking. Alcohol can inhibit the central nervous system (CNS), cause respiratory depression and, eventually, lead to death by asphyxia [12,13]. Autopsies of cases of death due to AAP lack specific anatomical–pathological findings, compared with other subjects within forensic medicine. Forensic scientists must, therefore, exclude other suspicious causes of death and combine auxiliary tests to reach the correct conclusion.

In forensic medicine, prior alcohol consumption and the quantity of alcohol intake are important for determination of responsibility of criminal and civil cases. It is important to judge death by AAP correctly. We assessed three fatal cases of AAP and, combined with a literature review, conducted a retrospective analysis and appraisal of AAP cases leading to death.

## Case presentation

### Case 1

**Figure 1.**
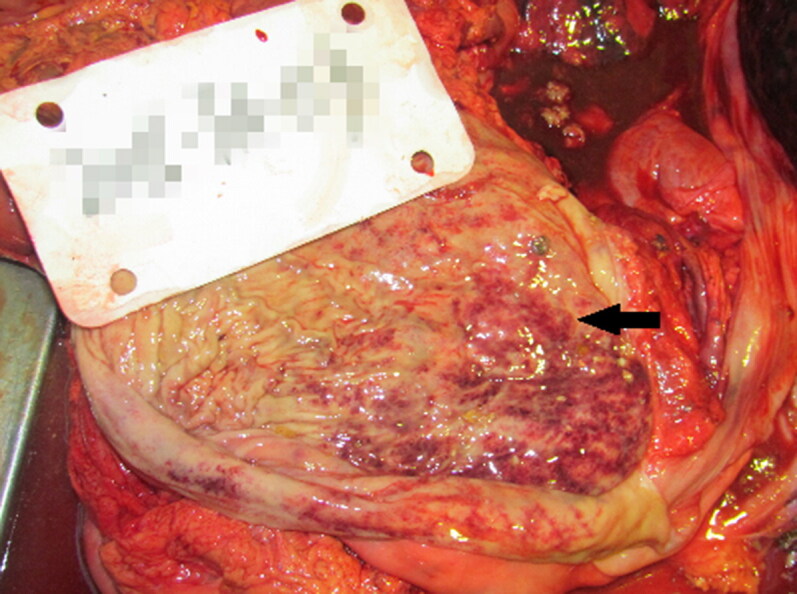
Light haemorrhage of the gastric mucosa (arrow).

A 44-year-old male was found deceased at home at 10 pm. After collection, his body was stored frozen. We conducted an autopsy 10 days after his death. External examination showed that his face, lips and nails were marked by cyanosis. A small rupture (0.3 cm × 0.3 cm) was found on his right occipitalis muscle. Internal examination revealed a thickened intima and lipid deposition in the left anterior descending coronary artery (LADCA), type-I stenosis of the LADCA lumen, cardiac and pulmonary interstitial congestion, severe pneumo-oedema and light haemorrhage of the gastric mucosa (Figure 1). The pathological diagnosis was: (1) severe pneumo-oedema, (2) cardiac and pulmonary congestion, and (3) small rupture of the right occipitalis muscle (0.3 cm × 0.3 cm).

Central blood (without a fluoride preservative) and gastric contents were taken for toxicology testing, and samples were stored at 4 °C before testing. The BAC was estimated by headspace gas chromatography using a method described previously [14]. A total of 0.10 mL of blood was diluted with tert-butanol (40.0 µg/mL; 0.50 mL as the internal standard). The same method was employed for the two cases described below. Toxicology testing showed that the BAC was 5.28 mg/mL. Common toxins, including hypnotic sedative drugs, insecticides and tetramine, were not detected in the submitted samples of blood or gastric contents. After investigation, we found that this man had consumed a whole bottle (500 mL) of spirit (ethanol content: 52%) at noon on the same day that he died. Thus, we concluded that this man’s cause of death was consistent with AAP.

### Case 2

The deceased was a 27-year-old female. She drank ∼450 mL of Hennessy cognac (ethanol content: 40%) with some people over the span of one night, and then went with other people to a restaurant, where she drank ∼150 mL of spirit (ethanol content: 35%). Subsequently, the woman slipped into a coma and was sent to a hospital. The attending physicians gave her an intravenous drip, but the woman died. The clinical diagnosis noted that the woman had drunk alcohol before slipping into a coma, her breathing and heartbeat had stopped, and her carotid pulse had disappeared by the time the ambulance arrived. After collection, her body was stored frozen.

**Figure 2.**
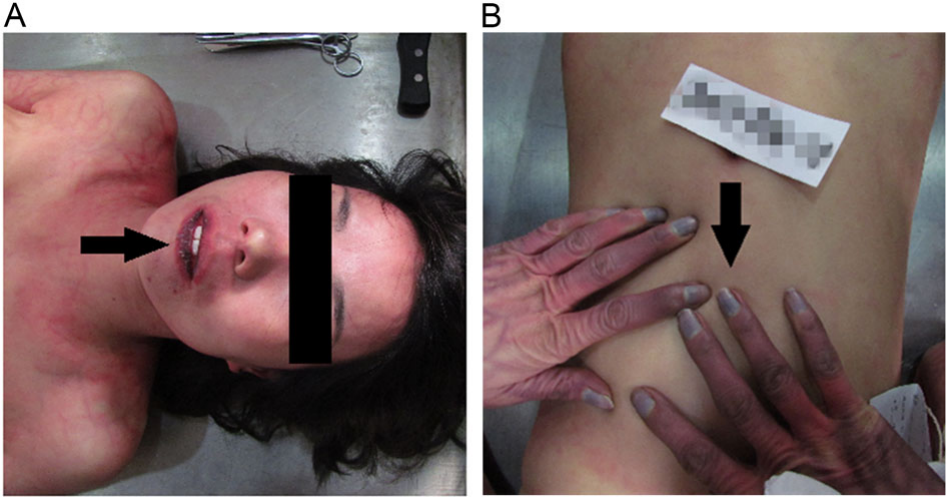
Lips (A) and nails of both hands (B) were marked by cyanosis.

We conducted an autopsy 17 days after her death. External examination showed that the face, lips and nails of both hands were marked by cyanosis (Figure 2). There was an injection-needle mark on the dorsum of her left hand which was set during medical treatment. Internal examination revealed pale-red oedematous fluid in some alveolar spaces. The pathological diagnosis was pneumo-oedema due to alcohol consumption. Central blood (without a fluoride preservative) was taken for toxicology and stored at 4 °C before tests. Toxicology revealed the BAC to be 3.33 mg/mL. Blood tests (monoclonal antibody board test) for morphine, pethidine, cocaine, marijuana, ketamine, methadone, amphetamine and methamphetamine were negative. Thus, we concluded that the woman’s cause of death was consistent with AAP.

### Case 3

A 19-year-old male who had been healthy was found in an abnormal state at ∼10 pm after drinking two paper cups of spirit (ethanol content: 36%) and a paper cup of beer (ethanol content: 8%) at a party. The capacity of each paper cup was ∼250 mL. Without too much delay, he was sent to a hospital, but efforts to revive him proved futile. After collection, his body was stored frozen.

**Figure 3.**
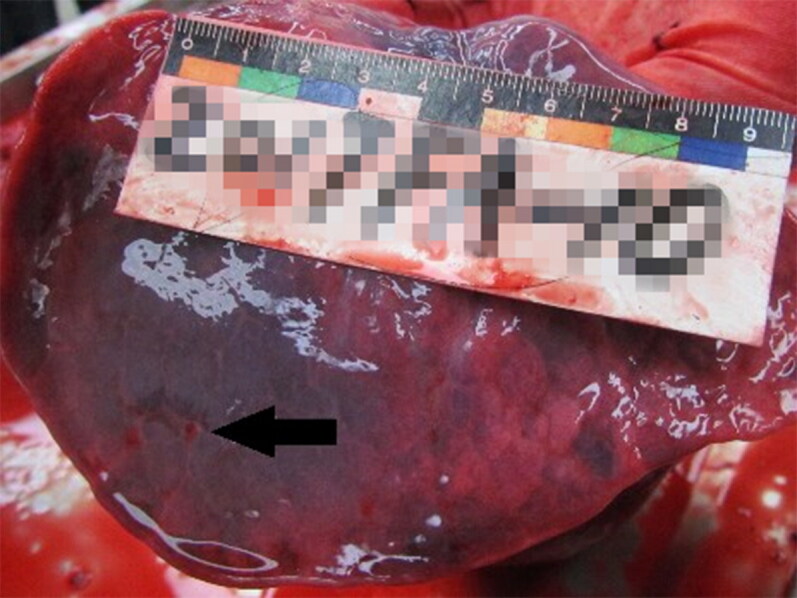
Small haemorrhagic spots on lung bases (arrow).

We conducted an autopsy 18 days after his death. External examination showed his lips and nails to be marked with cyanosis. There was an injection needle mark on the dorsum of his left hand (due to insertion of an intravenous drip at the hospital). Internal examination revealed some meaningful signs. The cut surface of the lungs showed congestion, and there were some small haemorrhagic spots on the lung bases (Figure 3). Most of the alveolar space was filled with pale-red oedematous fluid. The schistose regions of alveolar spaces were filled with red blood cells. Blood capillaries of alveolar walls were ectatic and congestive, and pulmonary interstitial congestion was present. A few haemorrhagic spots were observed on the schistose regions of the fundus gastric mucosa. A high level of congestion was found in the blood vessels of the gastric mucosa. Subarachnoid and cerebral parenchymal vascular congestion were also documented. The pathological diagnosis was: (1) pulmonary congestion, pneumo-oedema and localized pneumorrhagia, (2) focal haemorrhage of the gastric mucosa, and (3) cerebral haemorrhage and encephaloedema. Central blood (without a fluoride preservative), urine and gastric contents were taken for toxicology testing, and all samples were stored at 4 °C before testing. Toxicology testing revealed the BAC to be 3.78 mg/mL. Urinary tests for opioids, amphetamines and ketamine were negative. Hypnotic sedative drugs, insecticides or tetramine were not detected in his blood, urine or gastric contents. After investigation, we found that this man drank alcohol heavily at the party and had not slept well for 2 days previously. Thus, we concluded that the cause of this man’s death was consistent with AAP.

## Discussion and conclusion

The relationship between CNS depression and alcohol intoxication has been demonstrated [13]. Hence, people who appear to die from AAP often have asphyxia-related features. The three cases reported in the present study exhibited some important signs of asphyxia, such as cyanosis of the lips and nails, pneumo-oedema, pulmonary congestion and alveolar spaces being filled with pale-red oedematous fluid under microscopic examination. In Case 1, there was some haemorrhage in the gastric mucosa. In Case 3, we found some haemorrhagic spots on the schistose regions of the fundus gastric mucosa. These features were caused by acute injury to the gastric mucosa due to excessive consumption of alcohol. Alcohol can stimulate the gastric mucosal epithelium and submucosal vessels directly, and can change hormone levels in the gastrointestinal tract by mediating inflammation and thereby aggravating gastric mucosal injury [15,16].

The autopsy results of AAP-related death are not specific, so we should exclude other causes of death, such as sudden death, mechanical injury, mechanical asphyxia or electrocution. Alcohol intake is a risk factor for sudden cardiac death [17]. In Case 1, we found a thickened intima, lipid deposition in the LADCA and type-I stenosis in the LADCA lumen, but the latter was too light (Grade I: lumen area decreased by 1%–25%) to interrupt the cardiac blood supply, let alone cause cardiac arrest. We did not find pathological changes leading to sudden cardiac death either, so we ruled out this possibility. In addition, although a rupture (0.3 cm × 0.3 cm) was found on the right occipitalis muscle, its range was limited, and we did not detect a skull fracture or obvious intracranial haemorrhage. Therefore, we could exclude death by craniocerebral injury. Alcohol intoxication can result in vomiting, food regurgitation and paralysis of the pharyngeal reflex, which make it easier to die from asphyxia *via* aspiration of gastric contents [18]. According to the anatomy of asphyxia-related death caused by aspiration, vomit in the trachea, bronchi and bronchioles may be found. In the three cases reported here, there was no blockage in the trachea, bronchi or bronchioles due to foreign bodies.

In addition to autopsy, we used auxiliary tests, including a BAC test and other routine toxicology tests, to determine the cause of death. Through case investigations, it has been established that AAP-related death is often associated with a history of heavy drinking. Compared with chronic alcoholism, AAP shows a higher BAC but the acetone concentration is not increased remarkably. These findings may be related to alcohol-related damage to organs and tissues, chronic tolerance to alcohol, positional asphyxia/suffocation, hypoglycaemia or ketoacidosis [19].

Different authorities have different views on the lethal blood concentration associated with AAP, and range from 3.50 to 4.00 mg/mL [4,20]. The mean BAC in 175 fatal cases recorded by Heatley and Crane [21] was 3.55 mg/mL. As a result of differences between individuals, the lethal threshold of the BAC is undefined and seems to be lower than conventional acknowledgement [22]. Jones [23] stated that the BAC of a driver in Sweden was >5.00 mg/mL but he was alive. The lethal dose of alcohol is related to sex, age and genetic factors, though the speed of alcohol consumption, type of beverage and drinking habits can also exert influences. Li et al. [24] showed that, in AAP-related death, overweight drinkers showed more significant BAC levels for the heart and peripheral circulation than normal-weight drinkers. The BAC in the three cases in the present study was 5.28, 3.33 and 3.78 mg/mL, respectively. Because of the high BAC in Case 1 (5.28 mg/mL), there is little doubt that the cause of death was AAP. In Case 2, the deceased consumed a large amount of high-alcohol-concentration spirit, which may resulted in a decrease in alcohol tolerance. She had a history of coma before death, which confirmed that her BAC had reached a lethal concentration. In Case 3, the deceased was only 19 years of age. Compared with adults, adolescents are more sensitive and less tolerant to alcohol [13]. Further investigation revealed that the deceased drank quickly and had not slept for 2 days before the incident, which may have reduced his tolerance to alcohol. A history of intravenous-drip rescue was found in Cases 2 and 3, which may have led to the decline in the BAC. The relationship between alcohol intake and the BAC is expressed by the Widmark formula [25,26]:

$$A\left(g\right)=\mathrm{BAC}\left(\mathrm{mg}/g\right)\times \mathrm{BWt}\left(\mathrm{kg}\right)\times \mathrm{rho}‐\mathrm{factor}$$

where BAC represents blood-alcohol concentration in mass/mass unit; BWt represents the body weight (kg); *A* represents the quantity (g) of alcohol absorbed and distributed in all body fluids and tissues at the time the blood sample was taken; and the rho-factor differs in different individuals. This formula provides a preliminary inference of the BAC, and verifies the accuracy of the case investigation and BAC-test results.

Results of ethanol determination in specimens postmortem can be affected by several factors, of which putrefaction and diffusion of gastric contents postmortem are the most common [1]. Ensuring timely submission to the test after death and keeping corpses frozen to inhibit bacterial activity can reduce the interference of putrefaction postmortem. Currently, n-propanol is used as an internal standard. However, the theory has been invalidated that if the deceased did not have prior alcohol consumption, the ratio of alcohol/n-propanol in his/her blood should be <20 [14]. Therefore, the expected ratio of alcohol/n-propanol must be determined.

Ethyl glucuronide (EtG) and ethyl sulfate (EtS) are products of alcohol metabolism, and can be produced only by enzymatic metabolism from the body. Under some circumstances, analyses of these ethanol metabolites can be useful to estimate putrefaction postmortem. However, EtG has a long half-life and may be unstable at 30 °C or 40 °C if blood samples are stored without preservatives [14]. Therefore, EtG cannot show that alcohol consumption is related directly to the death, and is not suitable for highly putrefactive corpses.

The urine alcohol concentration (UAC) can help to analyze alcohol metabolism. The UAC/BAC ratio could help to ascertain if the absorption and distribution of alcohol in all fluids is complete. Usually, a low ratio (both mean and median, 1.18:1) suggests that alcohol has been absorbed and distributed completely [27].

Besides its watery nature, the vitreous humour is less affected by bacterial spread from the intestinal tract, which makes it undergo less putrefaction post-mortem and has less redistribution postmortem [1]. Therefore, it can be used as a substitute and reliable assessment if blood is deficient or if there is putrefaction or redistribution postmortem [28].

Our three cases were sent for autopsy and toxicology tests 10, 17 and 18 days after death but the bodies were frozen, and organ autolysis was not obvious according to histology. Thus, we consider the BAC results to be reliable. Usually, the liquor pericardii exhibits the highest alcohol concentration, followed by the left pulmonary vein, aorta, left heart, pulmonary artery, superior vena cava, inferior vena cava, right heart, right pulmonary vein and femoral vein, in decreasing order [29]. Therefore, taking femoral venous blood simultaneously for a BAC test may help reduce the error from diffusion of gastric contents.

In fatal cases of AAP, detection of other drugs is not infrequent, of which diazepam is predominant [30]. Alcohol can interact with other drugs and promote their toxicological effects. Studies have shown that alcohol consumption increases the risk of death for heroin users [31]. Methamphetamine and cocaine can enhance the toxicological effects of alcohol [32]. Meanwhile, the BAC decreases if the number of other drugs in blood increases, which has an impact on results [33]. There are cases of death due to alcohol combined with other poisons (e.g. injecting drugs while consuming alcohol). Indeed, some criminals taint alcoholic beverages to commit crimes. In China, there is a tradition of drinking medicated wine, which may contain toxic components such as venin or aconitine [34]. Therefore, for cases of death due to AAP, routine toxicology tests should be done to exclude the possibility of death by other poisons. There are many types of toxic substances, so some rare toxic substances may be omitted from routine toxicology analysis. Thus, it is necessary to combine the autopsy, case details, and the crime-scene investigation with toxicology tests to make a comprehensive analysis. In all three cases, routine toxicology tests were carried out, and we did not find other meaningful discoveries through case investigation and autopsy. Therefore, other poisons could be excluded from the causes of death.

In recent years, extensive studies have interpreted how alcohol-induced inhibition of the respiratory centres in the brainstem help explain the cause of AAP-related death. Acute exposure to alcohol can influence synaptic transmission, break the delicate balance between excitatory and inhibitory neurons in the CNS, and inhibit brain functions by altering cell membranes, ion channels, enzymes, neurotransmitter receptors, and the proteins involved in intracellular signal transduction [35,36]. In pre-synaptic signalling, the main function of alcohol is to increase the release of γ-aminobutyric acid (GABA). According to *in vivo* and *in vitro* studies, alcohol intake can increase the GABA concentration significantly in some brain regions (amygdala, hippocampus, brainstem) and cause a dose-dependent increase in GABA release in Purkinje cells [37,38]. Recent studies have suggested that release of intracellular calcium, protein kinase A (PKA), protein kinase C (PKC) and adenylate cyclase have roles in this increase in the GABA concentration [39]. In post-synaptic signalling, alcohol acts mainly on neurotransmitter receptors and affects their functions. Alcohol has a non-competitive inhibitory effect on *N*-methyl-d-aspartic acid receptors, which are highly sensitive to acute exposure to alcohol by inhibiting calcium influx [40]. Furthermore, alcohol can strengthen the affinity between GABA and GABA receptors, increase influx of chloride ions, and enhance the long-term depression of GABA_A_ receptors [41]. Experiments have shown that PKC is necessary for the alcohol-induced potentiation of GABAergic function, and that PKCσ plays an important part in activation of σ-subunit-containing GABA_A_ receptors [41,42]. Alcohol can also act directly on the hydrophobic domain of 5-hydroxytryptamine-3 (5-HT_3_) receptors, where it makes the opening of these channels more stable, potentiates the function of 5-HT_3_ receptors and, eventually, enhances CNS depression [43,44].

As stated above, the forensic appraisal of AAP-related death should pay attention to four main features. First, the case information must be considered, detailed questioning of witnesses should be conducted, and the scene should be surveyed carefully. People who have died due to AAP often have a history of alcohol abuse. Empty or partially empty bottles of alcoholic beverages — or even vomit — can be found at the scene of death. Investigators should pay attention to and collect suspicious items, such as physical evidence on the body and clothing of the deceased. If there is suspicion that the deceased had consumed adulterated wine or medicinal liquor or had been poisoned with toxic substances other than an alcoholic beverage, all of the edible food, drink, and vomit should be sealed and sent for testing. Data from video surveillance can, in some cases, help clarify the activities of the deceased and other details. The second feature is a complete and detailed autopsy. Suspicious injuries or abnormal body conditions/phenomena postmortem should be looked for. AAP-related death exhibits asphyxiation and excludes other causes of death, such as mechanical injury, mechanical asphyxia or electrocution. The third feature is common forensic toxicology testing (e.g. BAC). To eliminate interference by diffusion of gastric contents, blood from the femoral vein should be taken for toxicology testing. Samples of urine and the vitreous humour can be taken simultaneously, if necessary. For highly putrefactive corpses, extraction of the vitreous humour can be attempted for testing [45]. Meanwhile, routine toxicology tests should be carried out and combined with the case investigation and autopsy. The toxicological effects of different drugs and their blood concentration should be analyzed to determine the true cause of death and the potential role of alcohol. The final feature is a comprehensive analysis of various factors. When considering deaths after alcohol intake, investigators should not simply classify these cases as AAP-related death based on their first impressions, but instead should exclude other suspicious factors to ensure a strict and accurate forensic appraisal.
